# Ultralow Prostate-Specific Antigen (PSA) Levels and Improved Oncological Outcomes in Metastatic Hormone-Sensitive Prostate Cancer (mHSPC) Patients Treated with Apalutamide: A Real-World Multicentre Study

**DOI:** 10.3390/jcm13206221

**Published:** 2024-10-18

**Authors:** Alicia López-Abad, Mario Belmonte, Miguel Ramírez Backhaus, Gerardo Server Gómez, Enrique Cao Avellaneda, Cristóbal Moreno Alarcón, Pedro López Cubillana, Pablo Yago Giménez, Pedro de Pablos Rodríguez, María José Juan Fita, Miguel Ángel Climent Durán, Iris Guardiola Ruiz, Natalia Vidal Crespo, Juan Moreno Avilés, Pablo Luis Guzmán Martínez-Valls, Pedro Ángel López González

**Affiliations:** 1 Department of Urology, Virgen de la Arrixaca Hospital, El Palmar, 30120 Murcia, Spain; 2 Department of Experimental and Clinical Medicine, University of Florence, 50121 Florence, Italy; 3 Department of Urology, Instituto Valenciano de Oncología (IVO), 46009 Valencia, Spain; 4 Department of Urology, Santa Lucía Hospital, 30202 Cartagena, Murcia, Spain

**Keywords:** prostate cancer, metastatic volume, metastatic hormone-sensitive prostate cancer, PSA levels, ultralow PSA

## Abstract

**Background/Objectives:** Androgen receptor-targeted agents have significantly improved the prognosis of metastatic hormone-sensitive prostate cancer (mHSPC). Prostate-specific antigen (PSA) levels are key prognostic markers, with rapid and deep reductions associated with better outcomes. This study aims to assess the association between the new PSA cut-offs and survival in mHSPC patients treated with Apalutamide. **Methods:** We conducted a multicentre, retrospective analysis of mHSPC patients treated with Apalutamide between March 2021 and January 2023. Overall survival (OS) and radiographic progression-free survival (rFPS) were analyzed and stratified by the following PSA ranges: <0.02 ng/mL (ultralow), 0.02–0.2 ng/mL, and >0.2 ng/mL. Cox regression was applied to identify variables associated with OS and rPFS. **Results**: Among 193 patients, 34.2% had de novo mHSPC, with the majority classified as M1b. A total of 58.2% (110) of our cohort achieved ultralow PSA levels, with 20.6% between 0.02 and 0.2 ng/mL, and 21.2% of PSA levels > 0.2 ng/mL. Most patients reached ultralow PSA within six months. Low-volume, metachronous, and M1a subgroups displayed a higher prevalence of patients reaching ultralow PSA levels. At 18 months, OS was 100% in the ultralow PSA group, 94.4% for the 0.02–0.2 ng/mL group, and 67.7% in the >0.2 ng/mL group. Similarly, rPFS at 18 months was 100%, 93.5%, and 50.7%, respectively. Cox regression revealed that both ultralow PSA levels and ISUP grade had a significant impact on OS (HR of 8.256 and 0.164, respectively). For rPFS, only ultralow PSA levels had a significant impact (HR = 0.085). **Conclusions**: This real-world study of mHSPC patients treated with Apalutamide plus ADT revealed that achieving ultralow PSA levels is strongly associated with better oncological outcomes.

## 1. Introduction

Androgen deprivation therapy (ADT) has been the primary treatment for metastatic prostate cancer for decades [1]. However, several trials have shown clear and non-negligible benefits in disease progression and survival of metastatic hormone-sensitive prostate cancer (mHSPC) patients when ADT therapy was combined with the new androgen receptor pathway inhibitors (ARPI) such as Abiraterone, Apalutamide, Enzalutamide, Darolutamide [2,3,4,5,6], and/or chemotherapy (Docetaxel) [7,8]. For this reason, nowadays the European Association of Urology Guidelines strongly recommend combination therapy for the treatment of mHSPC [9]. Yet, the most appropriate combination of ADT and ARPI still has to be established.

The choice of the optimal treatment should be tailored based on the volume of metastasis, according to CHAARTED or LATITUDE criteria, the timing of metastasis presentation, and the patient’s specific features [5,10,11]. Specifically, the TITAN study demonstrated that Apalutamide, when combined with ADT, provided robust improvements in overall survival (OS) and radiographic progression-free survival (rPFS) to patients with mHSPC, regardless of time or volume of disease [3]. A recent post-hoc analysis of the TITAN assessing PSA kinetics demonstrated that Apalutamide allowed more patients to achieve a higher rate of PSA decline and, among these, those who achieved a rapid (<3 months) and deep (<0.2 ng/mL) PSA reduction were the ones who received the most benefits in terms of disease progression and survival [12].

Recently, we published our results from a multicentre study of patients with mHSPC treated with Apalutamide plus ADT, confirming the benefits found in the TITAN study and the relationship between the PSA kinetics and oncological outcomes that emerged from the aforementioned analysis [13].

With the recent development of ultrasensitive assays, it has become increasingly common to detect PSA levels below 0.2 ng/mL. Within the subset of mHSPC patients with PSA below this threshold, there might be underlying heterogeneity that could potentially conceal different outcomes. In this post hoc analysis of our previous study, we aim to expand the definition of deep PSA response and assess the possible relationship existing between even lower levels of PSA (<0.02 ng/mL or between 0.2 and 0.02 ng/mL) achieved after therapy with ADT plus Apalutamide and patients’ outcomes.

## 2. Materials and Methods

### 2.1. Study Design and Participants

Following approval from the Ethics Committee, this study analyzed a cohort of patients diagnosed with mHSPC in a retrospective, consecutive manner from March 2021 to January 2023. Participants were recruited from three leading medical institutions in Spain: Virgen de la Arrixaca University Hospital (Murcia, Spain), Fundación Instituto Valenciano de Oncología (Valencia, Spain), and Santa Lucía Hospital (Cartagena, Spain). The diagnosis of mHSPC followed the CHAARTED criteria, with patients further categorized by metastasis volume and timing, using the classifications proposed by Francini and Gravis [10,11].

### 2.2. Data Acquisition

Patient data were collected retrospectively from medical records, with the last follow-up until January 2023 or the patient’s death, whichever occurred first. The data were categorized into three key areas: baseline characteristics, post-Apalutamide data, and survival outcomes.

- Baseline characteristics included patient demographic (age), comorbidities (such as diabetes mellitus, ischemic cardiomyopathy, hypothyroidism, severe hypertension, anticoagulant medication use, severe renal failure, cardiac insufficiency and cognitive impairment), Eastern Cooperative Oncology Group Performance Status (ECOG PS), PSA levels at diagnosis, ISUP grade, history previous local prostate treatment (radical prostatectomy, radiotherapy, or both), as well as detailed information on metastatic burden, including the volume and location of metastases.
- After Apalutamide initiation, PSA reduction was assessed at 1, 3, 6, 12, and 18 months. PSA levels were categorized by predefined PSA ranges: >0.2, 0.02–0.2, and <0.02 ng/mL.
- Survival outcomes, including OS and rPFS, were analyzed according to the established PSA ranges. Time-to-event data were recorded in months, calculated from the date of Apalutamide initiation to either the event (death or radiographic progression) or the last follow-up.

### 2.3. Statistical Analysis

A comprehensive descriptive analysis was performed to examine patients’ baseline characteristics and PSA response. Categorical variables, such as patient demographics and comorbidities, were expressed as frequencies and percentages, while continuous variables (e.g., PSA levels, age) were summarized using medians and interquartile ranges to account for potential skewness in data distribution.

Survival analysis was conducted using the Kaplan–Meier method to OS and rPFS rates, with corresponding survival curves generated for different PSA decline categories. Predefined PSA ranges were assessed at landmark times of 1, 3, 6, 12, and 18 months following Apalutamide initiation.

To further explore the association between clinical variables and survival outcomes, Cox proportional hazards regression was performed to identify independent predictors of OS and rPFS. The proportional hazards assumption was checked for each model, and hazard ratios (HRs) with 95% confidence intervals (CIs) were reported.

All statistical tests were two-sided, with significance set at *p* < 0.05. SPSS for Windows version 25.0 was used to analyze data and generate graphical representations of survival curves.

## 3. Results

### 3.1. Patients’ Characteristics

In our retrospective study, we analyzed a cohort of 193 patients diagnosed with mHSPC from three medical centres in Spain. The median age of the patients was 72 years, with diabetes mellitus and hypertension being the most common comorbidities, affecting 30.3% and 27.3% of the patients, respectively. Most patients (65.5%) had an ECOG PS score of 0.

At the time of diagnosis, the median PSA level was 16 ng/mL, with a wide range from 2.5 to 1410 ng/mL. A total of 52.8% of our cohort of patients had a Gleason score below 8, and 34.2% were diagnosed with synchronous mHSPC. Regarding the extent of metastasis disease, 59.1% of patients were classified as M1b. A more comprehensive breakdown of the patients’ characteristics is provided in Table 1.

### 3.2. PSA Decline

Following the initiation of Apalutamide treatment, 58.2% (110 patients) achieved PSA levels below 0.02 ng/mL, 20.6% (39) reached PSA levels between 0.02 and 0.2 ng/mL, and 21.2% (40) PSA levels over 0.2 ng/mL. 

**Figure 1:**
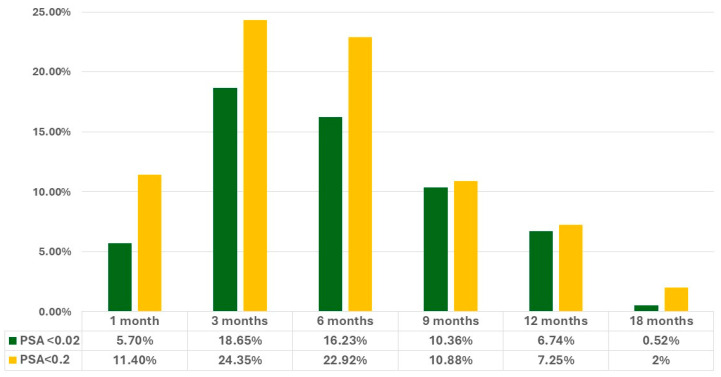
Incidence of PSA < 0.2 ng/mL and ultralow PSA (<0.02 ng/mL) achieved in the cohort at various time points during the follow-up.

The majority of patients who reached PSA levels below 0.2 mg/mL achieved this threshold within six months of starting Apalutamide treatment, with a mean time of 5.5 months and a median time of 6 months. For those achieving ultralow PSA levels (<0.02 ng/mL), the mean time was 5.8 months, with a median time of 6 months. Figure 1 illustrates the percentage of the cohort that reached PSA levels below 0.2 ng/mL and ultralow PSA levels during the follow-up period.

**Figure 2:**
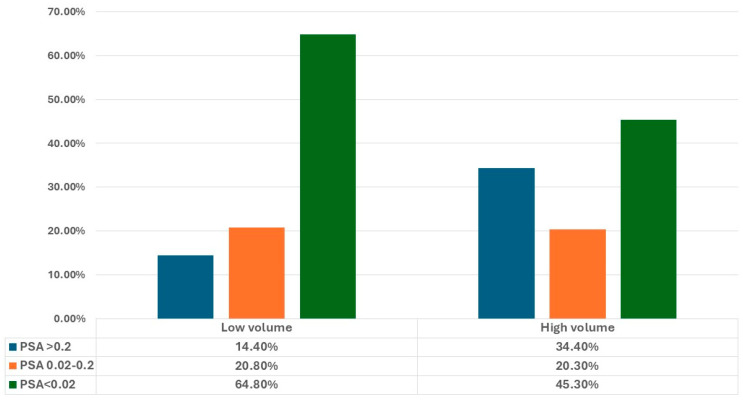
PSA ranges achieved in our cohort, stratified by metastasis volume (high volume vs. low volume).

**Figure 3:**
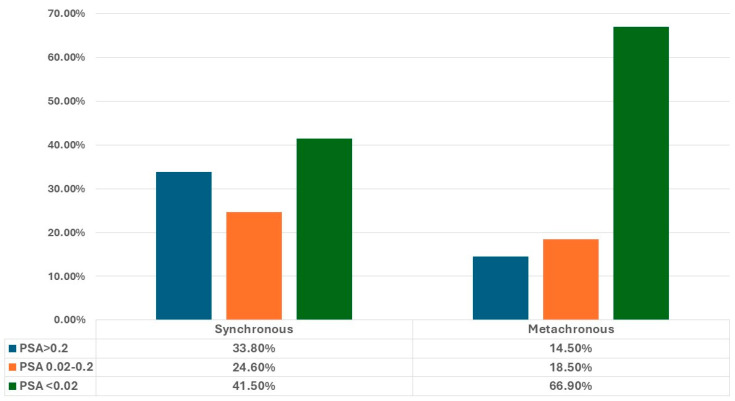
PSA ranges achieved in our cohort, stratified by time of presentation (synchronous vs. metachronous).

PSA decline was then analyzed across groups categorized by metastasis volume, timing, and “M” staging. Notably, the low-volume and metachronous groups displayed a higher prevalence of patients achieving PSA levels below 0.02 ng/mL (64.8% and 66.9%, respectively) compared to 45.3% in the high-volume group (*p* = 0.005) and 41.5% in the synchronous group (*p* = 0.001) (Figure 2 and Figure 3).

**Figure 4:**
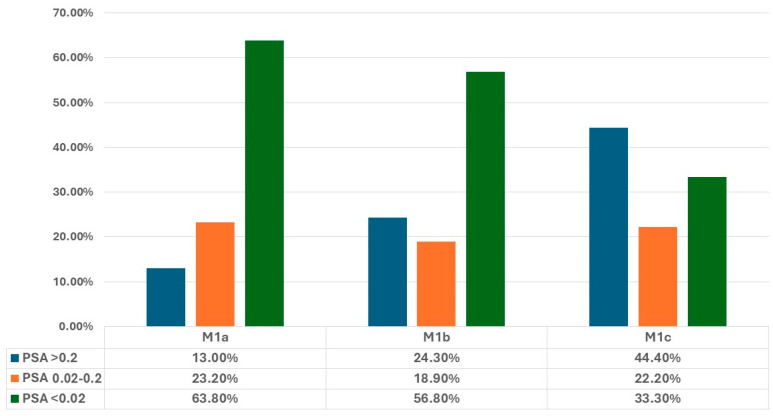
PSA ranges achieved in our cohort, stratified by the metastasis localization (“M” staging: M1a, M1b, and M1c).

When focusing on metastasis type, PSA < 0.02 was achieved by 63.8% of M1a patients, 56.8% of M1b patients, and 33.3% of M1c patients (*p* = 0.151) (Figure 4).

### 3.3. Overall Survival (OS) and Radiographic Progression-Free Survival (rPFS)

**Figure 5:**
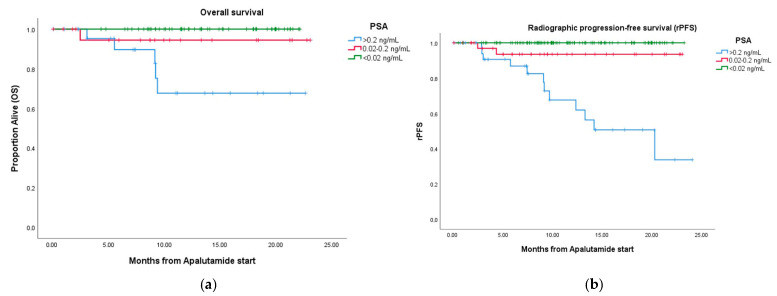
Kaplan–Meier survival curve for OS (**a**) and rPFS (**b**) by PSA ranges.

The OS rate at 18 months was 92.5%. When analyzed by PSA levels, OS was 67.7% for the PSA > 0.2 group, 94.4% for the PSA 0.02–0.2 group, and 100% for the PSA < 0.02 group (*p* = 0.000) (Figure 5a).

The 18-month rPFS rate was 88.9%. Using the same PSA categories, rPFS was 50.7% for the PSA > 0.2 group, 93.5% for the PSA 0.02–0.2 group, and 100% for the PSA < 0.02 group (*p* = 0.000) (Figure 5b).

Additionally, Cox regression was performed to analyze the relationship between OS and rPFS and specific variables, including metastasis volume, timing, “M” staging, PSA ranges, and ISUP grade. Both ISUP grade and ultralow PSA levels had a significant impact on OS, with a HR of 8.256 (CI 95% 1.679–40.598, *p* = 0.009) and 0.164 (CI 0.033–0.816, *p* = 0.0027), respectively. For rPFS, only ultralow PSA levels had a significant impact, with a HR of 0.089 (CI 95% 0.026–0.308, *p* = 0.000).

## 4. Discussion 

Combination therapy for mHSPC is the current standard of care nowadays, with several clinical trials demonstrating improved outcomes when compared to ADT monotherapy. As emerged from a recent meta-analysis, any of the new ARPI (Abiraterone, Enzalutamide, Apalutamide, Darolutamide) has shown a survival benefit in mHSPC when associated with ADT [14]. These beneficial effects have been demonstrated to be even more pronounced when a rapid and deep PSA decline (defined historically as <0.2 ng/mL) occurs after therapy initiation [15].

It is quite clear that the lower the PSA, the better the outcomes. However, with the development of the ultrasensitive PSA assay, the range of PSA < 0.2 ng/mL may not be as small as it looks. For instance, reaching ultralow levels of PSA (<0.01 ng/mL) after radical prostatectomy is associated with an 86.1% recurrence-free survival rate at 11 years, compared to 48.9% for PSA levels greater than 0.01 ng/mL [16]. Similarly, biochemical relapse rates are strongly associated with the PSA nadir following radical prostatectomy, with the lowest relapse rate associated with PSA values < 0.01 ng/mL [17].

However, the optimal PSA nadir (defined as the lowest PSA value measured after treatment of mHSPC patient) remains a subject of ongoing investigation. Various studies have explored different PSA cut-offs, including <0.2 ng/mL, <0.05 ng/mL, and ≤0.02 ng/mL, consistently showing that achieving a PSA nadir of <0.2 ng/mL—and even more, <0.02 ng/mL—is associated with better oncological outcomes [18].

Focusing on the combination of Apalutamide and ADT in mHSPC, a recent post-hoc analysis of the TITAN study divided PSA levels under 0.2 ng/mL into two groups: ultralow one (UL1), with PSA levels between 0.02 and 0.2 ng/mL, and ultralow two (UL2), with PSA levels ≤ 0.02 ng/mL. Compared to ADT plus placebo, more patients in the Apalutamide group achieved UL1 and UL2 at 3 months (38% and 23%), and it was associated with significantly longer rPFS, OS, time to PSA progression, and time to castration resistance, especially in the UL2 groups. The same benefits persisted at the 6-month landmark [19].

Additionally, Wenzel et al. [18] observed that patients who achieved PSA nadir of ≤0.02 ng/mL with Apalutamide treatment had the best outcomes in terms of time to castration resistance and OS rates. Furthermore, when comparing Apalutamide and Abiraterone treatment in mHSPC, they found that a significantly higher proportion of patients achieved a PSA nadir ≤ 0.02 ng/mL with Apalutamide (40% vs 8.8%) [20].

In our cohort of 193 patients, 78.8% achieved PSA levels below 0.2 ng/mL. We successively expanded the definition of low PSA and stratified patients in two groups, as previously mentioned: 58.2% of patients achieved ultralow PSA levels (<0.02 ng/mL), while 20.6% achieved low PSA levels (between 0.02 and 0.2 ng/mL) by the 18-month landmark. Assessing the kinetics of PSA response, 58.67% of patients reached PSA levels below 0.2 ng/mL and 40.58% of patients reached ultralow PSA levels within the first 6 months of follow-up. The median time to reach these thresholds was 6 months in both groups (Figure 1).

These notably high response rates align with the PSA kinetics analysis from the TITAN study, which assessed PSA response at 3, 6, and 12 months. In TITAN, 38% of patients achieved low PSA levels and 23% achieved ultralow levels by 3 months. However, the proportion of patients achieving ultralow PSA level increased rapidly and by the 12-month mark, this group was twice the size of the low PSA group. For this reason, among patients with a PSA response < 0.2 ng/mL, a non-negligible fraction may still reach PSA levels < 0.02 ng/mL at later stages of continued therapy with Apalutamide, potentially gaining the full therapeutic benefits [19]. Similarly, in our cohort, we found a small number of patients who reached these defined thresholds after 18 months of treatment with ADT plus Apalutamide.

In our previous study, we demonstrated the benefits of patients achieving PSA level < 0.2 ng/mL [13]. However, the data that emerged from this new analysis show that the most favourable benefits in OS concern patients achieving PSA < 0.02 ng/mL. Wenzel et al. [18] also found that reaching a PSA nadir < 0.02 ng/mL at any point during ADT plus Apalutamide treatment was associated with statistically significant improvement in OS when compared to patients with PSA levels above 0.2 ng/mL or between 0.02 and 0.2 ng/mL. Notably, the median time to reach the nadir aligns with the findings from our cohort, specifically between 3 to 6 months. The TITAN analysis similarly observed the most significant improvement in OS in patients who reached PSA < 0.02 ng/mL at 3 months, when compared to those with PSA > 0.2 ng/mL. These benefits remained significant at longer follow-ups [19].

Similar findings were observed for rPFS and time to metastasis. In our cohort, none of the patients who achieved ultralow PSA levels at any point during therapy experienced radiographical disease progression. The TITAN analysis further confirmed that the most significant benefits in rPFS—time to castration-resistant prostate cancer and PSA progression—were observed in patients who reached this nadir at 3 months, compared to those with PSA > 0.2 ng/mL [19].

Although we did not assess the relationship between OS or rPFS and the timing of achieving low or ultralow PSA responses, our findings are noteworthy. In fact, all the patients who reached ultralow PSA levels at any point during their therapy were alive and with no radiographic progression, regardless of when they achieved their nadir.

However, we found statistically significant differences in ultralow PSA achievement rates among mHSPC patients stratified by metastasis volume and timing. Specifically, patients in the low-volume and metachronous groups had higher ultralow PSA achievement rates (64.8% and 66.9%, respectively) compared to those in the high-volume and de novo groups (45.3% and 41.5%, respectively). Similarly, Wenzel et al. [18] reported the highest rates of PSA nadir < 0.02 ng/mL in patients with low volume and metachronous disease. Despite the lower chances of achieving ultralow PSA levels, patients with high-volume and de novo disease are still likely to reach a decline of PSA to levels between 0.2 and 0.02. As demonstrated by the post-hoc analysis of the TITAN study, these patients with PSA between the aforementioned thresholds still showed significant OS and rPFS when compared with those with PSA > 0.2 ng/mL [19].

Nonetheless, our study has limitations that should be considered when interpreting the results. The bias determined by the retrospective, multicentre, and real-world nature of the study might be significant. Additionally, our cohort featured a relatively smaller proportion of de novo and high-volume mHSPC patients compared to the TITAN trial. Furthermore, unlike our cohort, the Apalutamide trial did not encompass mHSPC M1a patients. However, the strength of these encouraging results lies in our population, which consists of relatively numerous unselected patients with mHSPC drawn from three tertiary Spanish medical centres with extended follow-up.

## 5. Conclusions

The addition of Apalutamide to ADT monotherapy for the treatment of mHSPC is associated with higher achievement rates of ultralow PSA response. Moreover, reaching the threshold of PSA levels < 0.02 ng/mL at any point during the combination therapy confers robust, significant, and long-term benefits in OS and rPFS. These benefits persist regardless of the different and heterogeneous subgroups of mHSPC patients. Further comparative studies are needed to identify the optimal tailored therapy for this population.

## Figures and Tables

**Table 1:** Overview of the main characteristics of the included patients [13].

Variable		
**Age (median)**	72 years (48–89)	
**Follow-up (median)**	10 months (0–23)	
**Comorbidities**	Diabetes mellitus	30.3%
	Hypertension	27.3%
	Ischemic cardiopathy	6.1%
	Cardiac insufficiency	4.5%
	Anticoagulant treatment	7.6%
	Hypothyroidism	4.5%
	Cognitive impairment	0%
	Severe renal failure	1.5%
**ECOG**	0	65.5% (116)
	1	32.2% (57)
	2	2.3% (4)
**PSA at diagnosis (median)**	16 (2.5–1410)	
**ISUP grade**	1–3 (Gleason < 8)	52.8% (102)
	4–5 (Gleason ≥ 8)	47.2% (91)
**Metastasis timing**	Synchronous	34.2% (66)
	Metachronous	65.8% (127)
**Previous local treatment**	Radical prostatectomy (RP)	13% (25)
	Radiotherapy (Rt)	21.2% (41)
	RP + Rt	31.6% (61)
**Metastasis**	M1a	36.3% (70)
	M1b	59.1% (114)
	M1c	4.7% (9)
**Francini groups**	DN/LV	18.7% (36)
	DN/HV	15.5% (30)
	PD/LV	47.7% (92)
	PD/HV	18.1% (35)
**Bone metastasis**	Oligometastatic (≤3)	58.5%

DN: de novo or synchronous; LV: low volume; HV: high volume; PD: progressive disease or metachronous.

## Data Availability

The data supporting the findings of this study are accessible upon reasonable request to the corresponding author, subject to the Ethics Committee’s statement.

## Abbreviations

ADT	androgen deprivation therapy
ARPI	androgen receptor pathway inhibitors
ECOG PS	Eastern Cooperative Oncology Group Performance Status
HR	hazard ratio
mHSPC	metastatic hormone-sensitive prostate cancer
OS	overall survival
rPFS	radiographic progression-free survival
